# Correction: Charge engineering controls cooperative assembly and loading in protein host–guest complexes

**DOI:** 10.1039/d5tb90102b

**Published:** 2025-06-23

**Authors:** Zhiheng Wang, Dai-Bei Yang, Joshua A. Bulos, Rui Guo, Thomas Troxler, Sergei Vinogradov, Jeffery G. Saven, Ivan J. Dmochowski

**Affiliations:** a Department of Chemistry, University of Pennsylvania 231 S. 34th Street Philadelphia Pennsylvania 19104-6323 USA ivandmo@sas.upenn.edu; b Department of Biochemistry and Biophysics, Perelman School of Medicine, University of Pennsylvania Philadelphia Pennsylvania 19104 USA

## Abstract

Correction for ‘Charge engineering controls cooperative assembly and loading in protein host–guest complexes’ by Zhiheng Wang *et al.*, *J. Mater. Chem. B*, 2025, https://doi.org/10.1039/D5TB01202C.

The authors regret that an incorrect version of Fig. 5 was included in the originally published article. The correct version of Fig. 5 is shown below.

**Fig. 5:**
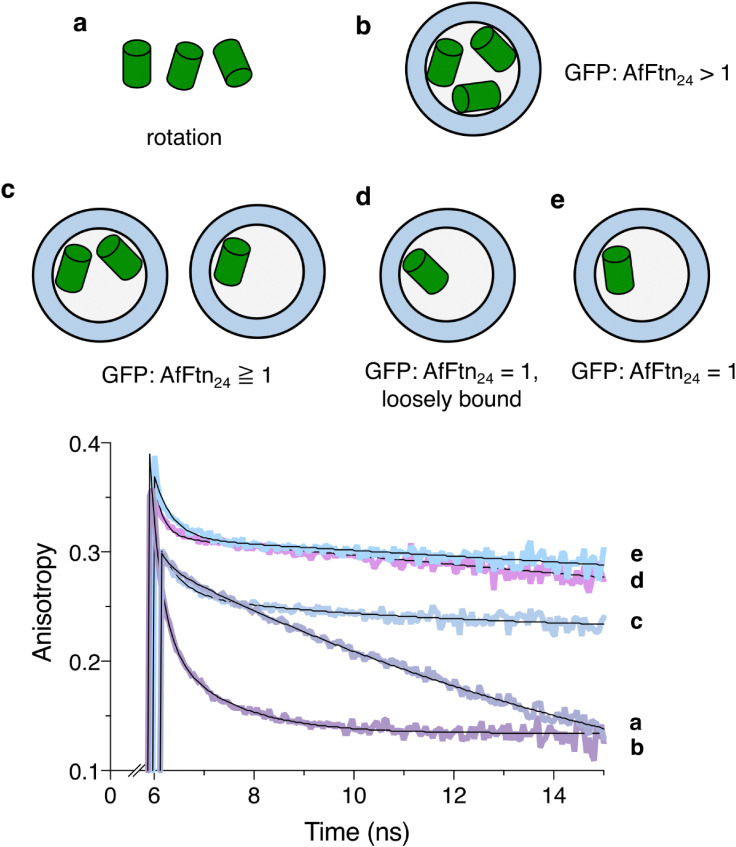
Time-resolved fluorescence anisotropy of AfFtn–GFP. (a) GFP(+36) in the low ionic strength buffer, pH 7.6. The observed decay time (12.1 ns) is consistent with the rotational diffusion of GFP. (b) SEC-purified AfFtn–GFP(+36) at pH 7.6. (c) SEC-purified AfFtn–GFP(+36) at pH 5.8. From the fits, three decay times were obtained and associated with instrument response, homoFRET, and rotation. (d) AfFtn–GFP(+36) assembly at pH 7.6, 500 mM NaCl. (e) AfFtn–GFP(+36) assembly at pH 7.6, 175 mM NaCl. In (d) and (e), both anisotropy decays were fit biexponentially with only instrument response and rotation diffusion of the assembly with no homoFRET observed. This suggested that a 12-to-1 complex was formed under both conditions. The *t* = 0 was set by the timing of the excitation laser pulse, and the data acquisition started around 6 ns. Each experiment was conducted in triplicate, and the sum files were plotted. In all samples, [GFP] = 1 μM. In each case, the anisotropy was fit with a multiexponential model equation (see Methods). Conditions and fitted fluorescence anisotropy parameters with uncertainties are summarized in ESI,† Table S1.

The Royal Society of Chemistry apologises for these errors and any consequent inconvenience to authors and readers.

